# Navigation versus fluoroscopy in minimalinvasive iliosacral screw placement

**DOI:** 10.1186/s13018-024-04669-0

**Published:** 2024-03-16

**Authors:** Max Prost, Roman Taday, Carl Christoph Paul Beyersdorf, David Latz, Joachim Windolf, Max Joseph Scheyerer, Markus Rafael Konieczny

**Affiliations:** 1 Department of Orthopedic and Trauma Surgery, Medical Faculty and University Hospital Düsseldorf, Heinrich-Heine-University Düsseldorf, Moorenstraße 5, 40225 Düsseldorf, Germany; 2 Department of Spine Surgery, Volmarstein Orthopedic Clinic, Volmarstein, Germany

**Keywords:** Navigation, Minimal invasiv surgery, Iliosacral screw placement, Osteoporotic fractures, Sacral insufficiency fracture

## Abstract

**Introduction:**

When needed operative treatment of sacral fractures is mostly performed with percutaneous iliosacral screw fixation. The advantage of navigation in insertion of pedicle screws already could be shown by former investigations. The aim of this investigation was now to analyze which influence iliosacral screw placement guided by navigation has on duration of surgery, radiation exposure and accuracy of screw placement compared to the technique guided by fluoroscopy.

**Methods:**

68 Consecutive patients with sacral fractures who have been treated by iliosacral screws were inclouded. Overall, 85 screws have been implanted in these patients. Beside of demographic data the duration of surgery, duration of radiation, dose of radiation and accuracy of screw placement were analyzed.

**Results:**

When iliosacral screw placement was guided by navigation instead of fluoroscopy the dose of radiation per inserted screw (155.0 cGy*cm^2^ vs. 469.4 cGy*cm^2^ *p* < 0.0001) as well as the duration of radiation use (84.8 s vs. 147.5 s *p* < 0.0001) were significantly lower. The use of navigation lead to a significant reduction of duration of surgery (39.0 min vs. 60.1 min *p* < 0.01). The placement of the screws showed a significantly higher accuracy when performed by navigation (0 misplaced screws vs 6 misplaced screws—*p* < 0.0001).

**Conclusion:**

Based on these results minimal invasive iliosacral screw placement guided by navigation seems to be a safe procedure, which leads to a reduced exposure to radiation for the patient and the surgeon, a reduced duration of surgery as well as a higher accuracy of screw placement.

## Introduction

Besides of traumatic fractures of the sacrum, there is an increasing incidence of osteoporotic fractures of the sacrum [1, 2]. Depending on the stability of the fracture and the level of pain there are conservative or operative treatment strategies for both types of fractures [3]. In many cases operative treatment is needed which is mainly performed by inserting iliosacral screws (ISS) [4, 5].

Placement of ISS can be performed as an open or as minimal invasive procedure (MIS). Due to better outcomes according to wound necrosis, infections and blood loss percutaneous and MIS procedures are preferred to open procedures [6, 7]. There are different techniques for the percutaneous insertion of ISS, among them ISS placement guided by fluoroscopy or guided by navigation [8, 9].

The advantage of applying navigation instead of fluoroscopy with regard to accuracy of screw placement, dose of radiation for the patient and for the surgical team has already been shown for insertion of pedicle screws by former investigations [10, 11].

Due to the fact, that spine surgeons are exposed to an up to 10 up to 50-fold higher dose of radiation than other non-spinal musculoskeletal surgeons’, it seems imperative that we should aim at a reduction of radiation exposure in our treatment methods [12–14].

There are former investigations which showed advantages in ISS placement guided by navigation instead of fluoroscopy with regard to accuracy of screw placement [9, 14, 15]. Present results on the impact on exposure to radiation are inconclusive. Some investigations state a higher dose of irradiation for the patient [9], some state a reduced dose of radiation [14, 15].

Due to this inconclusive data the aim of this investigation was to analyze the influence of navigation in ISS placement on duration of surgery, exposure to radiation for the patient and to the surgical team as well as the accuracy of screw placement compared to the fluoroscopy guided technique. Our hypothesis was, that navigation in ISS placement compared to screw placement guided by fluoroscopy leads to less exposure to radiation and a higher accuracy in screw placement without lengthening the operative procedure.

## Patients and methods

This study was performed as a single center study. We included patients who have been treated by MIS ISS placement in our department between 12/2014 and 05/2023. Patients who have been treated before 03/2021 were treated by MIS ISS placement guided by fluoroscopy, after 03/2021, ISS placement was performed guided by navigation. The patients in whom ISS placement was guided by fluoroscopy have been identified by diagnostic code and were included retrospectively. The patients in whom ISS placement was guided by navigation were included prospectively.

We excluded patients, who were treated for other musculoskeletal problems than the sacral fracture in the same surgery. Further, we excluded patients with an incomplete set of data. Patients who did not, or could not, agree to take part in the investigation were excluded, too.

Inclusion and exclusion criteria were summed up in Table 1.

**Table 1:** Inclusion and exclusion criteria

Inclusion and exclusion criteria	
Inclusion criteria	Exclusion criteria
Age over 18	Age under 18
Treatment by MIS ISS for sacral fractures MIS ISS placement guided by fluoroscopy was included retrospectively MIS ISS placement guided by navigation was included prospectively	Treatment for other musculoskeletal problems than the sacral fracture in the same surgery (e.g. spinal instrumentation or symphysis plating) Present implants in the area of the posterior pelvic ring before surgery
Complete set of data including pre- and postoperative CT scan and complete perioperative documentation of radiation use	Incomplete set of data
Written consent to participate	Missing consent to participate

Clinical and demographical data, duration of surgery, emitted dose and duration of radiation, screw length, accuracy of ISS placement and complications were recorded and analyzed. ISS were regarded as “incorrectly positioned” when there was a perforation of the ventral cortex of the sacrum or a penetration of the adjacent neuroforamen. Three orthopedic and trauma surgeons, each with more than 5 years of experience in the placement of ISS in a postoperative CT scan, assessed screw positioning. If assessment of an ISS differed between the investigators, the worst assessment was included in the study. The radiographs were analyzed by the IDS 7-PACS^®^-System (Sectra, Linköping, Sweden). In all patients the same C-arm (Arcadis; Siemens Healthineers, Forchheim, Germany) was used. In all patients, fluoroscopy was applied intermittently, not continuously. The MIS Screw-System from Axomed (Marquart Medizintechnik GmbH, Germany) with Screws with a diameter of 7.5 mm was used in all patients. All patients were treated by orthopedic and trauma surgeons with experience in pelvic surgery.

### ISS placement guided by navigation

The Navigation System ‘Kick’ (Brainlab, Munich, Germany) was applied when ISS placement was performed guided by navigation. The reference for the registration of the images was fixated to the anterior iliac crest by two 3.0 mm K-Wires. Then a 3D scan of the pelvis was performed. During that time the operative team left the operating room. After that subsequently, first placement of a guide wire and then insertion of the ISS was performed, guided by navigation. At the end, p.a. and lateral radiographs of the whole construct were taken.

### ISS placement guided by fluoroscopy

Insertion of the ISS was performed under fluoroscopic guidance [poster anterior, inlet view, outlet and lateral view]. All four types of radiographs were taken during the placement of the screws and at the end of the procedure to document the ISS positioning.

**Fig. 1:**
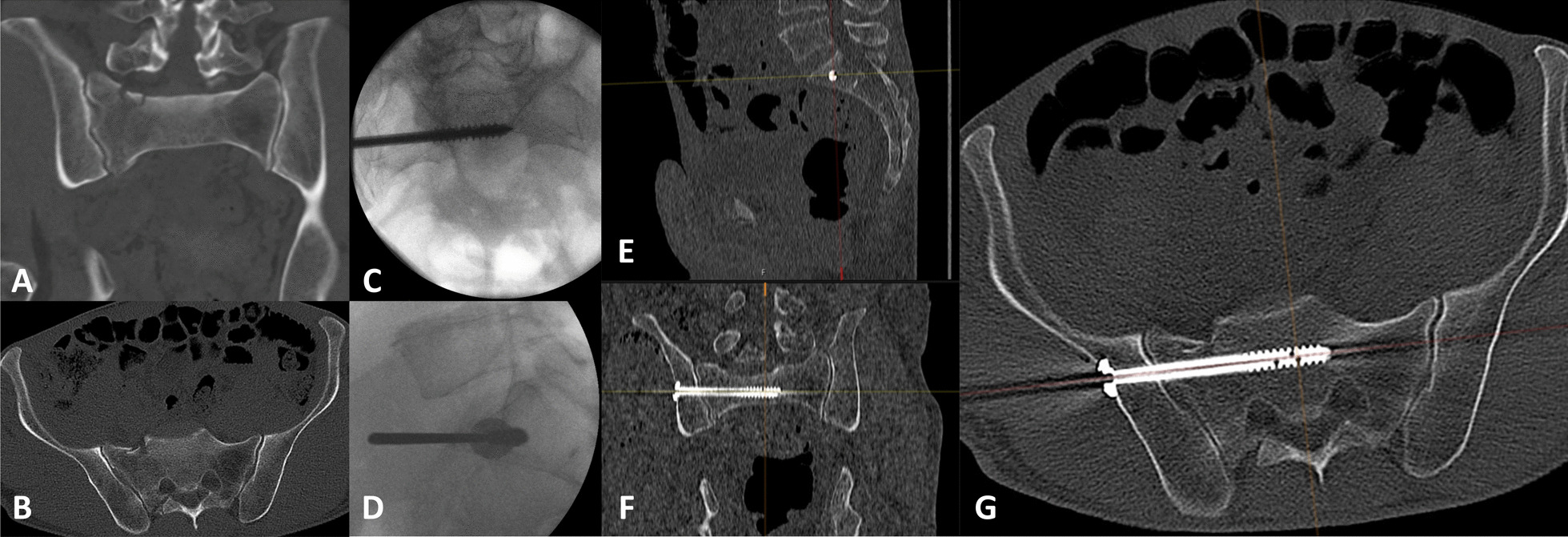
Exemplarily preoperative, intraoperative and postoperative radiographs of a patient treated by MIS ISS placement guided by navigation for a traumatic fracture of the sacrum. **A** and **B** shows the preoperative CT scan (**A** in frontal view, **B** in axial view). **C** and **D** shows p.a. (**C**) and lateral (**D**) intraoperative radiographs after the ISS placement guided by navigation with the guide wire still in situ. **E**–**G** shows screw positioning in the postoperative CT scan (**E** in sagittal, **F** in frontal view and **G** in axial view)

Preoperative, intraoperative and postoperative radiographs of a patient treated with MIS ISS placement guided by navigation were shown exemplary in Fig. 1.

### Assessment of radiation

Assessment of radiation was performed according to a primary described method by our study group [11]. Dose area product (DAP) has been retrieved from the automatically recorded protocol of the Arcadis Orbic 3D (Siemens) for all patients. Time of fluoroscopy of one 3D scan (used for navigation) was 60 s. We separately recorded DAP (cGy*cm^2^) with the surgical team in the operation theater (exposed to radiation) and with the surgical team not in the operation theater (not exposed to radiation) while the 3D scan was performed.

### Statistics

Statistical analysis was performed by SPSS^®^ 25 (IBM, Armonk, USA). Descriptive data are given as mean and standard error of mean (SEM). We tested all continuous variables for normal distribution by Kolmogorov–Smirnov tests. All variables showed no normal distribution. Thus, we performed Man-Whitney-U and Chi-Square tests.

This study was approved by the local ethics committee (Register number 2021-1422) and was conducted according to the revised Declaration of Helsinki.

## Results

We identified 121 patients who were treated operatively by ISS placement guided by fluoroscopy in our institution between 12/2014 and 03/2021 by diagnostic code. After application of the inclusion and exclusion criteria, we enrolled 46 of these patients to our investigation. Furthermore, prospectively we included 22 patients who were treated operatively by ISS placement guided by navigation since 03/2021.

Overall, 85 ISS have been implanted in these patients (guided by navigation n = 30; guided by fluoroscopy, n = 55). 39 patients were female (57.4%), 29 patients were male (42.7%). The average age at the time of surgery was 60.2 (2.7) years.

In the group in which ISS placement was performed with navigation 12 patients were female (54.5%), 10 patients were male (45.5%) and the average age at the time of surgery was 65.4 (4.4) years. In the group in which ISS placement was performed with fluoroscopy 27 patients were female (58.7%), 19 patients were male (41.3%) and the average age at the time of surgery was 57.6 (3.4) years. The gender and age distribution in the groups showed no significant difference (*p* > 0.05).

In 55 patients ISS placement was performed only unilateral (navigated n = 15, fluoroscopy n = 40) and in 13 patients ISS placement was necessary bilateral (navigated n = 7, fluoroscopy n = 6).

The average length of the perioperative hospital stay of the patients was 19.1 (1.3) days. In the navigated group it was 20.4 (2.8) days and in the fluoroscopy group it was 18.4 (1.5) days. The difference between the groups showed no significance (*p* > 0.05).

Length of the implanted ISS was in average 88.2 (1.3) mm. In the navigated group it was 89.2 (2.5) mm and in the fluoroscopy group it was 87.8 (1.5) mm. The difference between the groups showed no significance (*p* > 0.05).

When ISS placement was guided by navigation instead of fluoroscopy the dose of radiation for the patient per inserted screw was significant lower (*p* < 0.0001). See Table 2.

**Table 2:** Dose of radiation for the Patient in cGy*cm^2^ compared between the group with navigation use and the group with fluoroscopy use

Dose of radiation for the patient in cGy*cm^2^			
Navigation		Fluoroscopy	
Mean	SEM	Mean	SEM
155.0	90.5	469.4	301.5
*p* < 0.0001			

A further significant difference was seen in the duration of radiation use between the navigation and fluoroscopy group with advantages for navigation for the patient (*p* < 0.0001) and for the surgical team, who left the operation theater during the 3D scan (*p* < 0.0001). See Table 3.

**Table 3:** Duration of radiation use in seconds compared between the group with navigation use and the group with fluoroscopy use

Duration of radiation use in seconds							
Patient				OP team			
Navigation		Fluoroscopy		Navigation		Fluoroscopy	
Mean	SEM	Mean	SEM	Mean	SEM	Mean	SEM
84.5	42.4	147.5	53.6	27.5	12.4	147.5	53.6
*p* < 0.0001				*p* < 0.0001			

Separate analyzes for the surgeon and the patient

The use of navigation led to a significant reduction of duration of surgery [39.0 min (19.9) vs. 60.1 min (32.8) *p* < 0.01].

Placement of the ISS showed a significantly higher accuracy when performed by navigation [0 (0%) misplaced screws in navigated technique vs. 6 (10.9%) misplaced screws in fluoroscopic technique—*p* < 0.0001]. No patient from the navigation group needed revision surgery. From the 6 misplaced screws of the fluoroscopy group 4 screws needed surgical revision.

## Discussion

We performed a single center study with a retrospective and a prospective study arm in which we included 68 patients treated with MIS ISS placement.

Between the groups we analyzed there were no significant differences according to age and gender distribution. Furthermore, age and gender distribution of patients in our investigation are comparable with other investigations according to this topic [6–9, 14–22].

With regard to the screw length of the implanted ISS we found no significant difference between both groups. Current literature dealing with this topic gives no information about the length of the used screws. In our institution every patient weather ISS placing is performed guided by navigation or guided by fluoroscopy gets a preoperative CT scan of the pelvis in which screw position and screw length could be planned. Due to this preoperative planning the missing difference in length of the screws might have been expected.

Advantages with regard to the screw placement are discussed controversial in the current literature. On the one hand there are investigations which described no advantages with regard to screw placement when navigation is used and report comparable malpositioning rates between navigation and fluoroscopy. Kułakowski et al. [16] reported 2022 a rate of misplaced screws of 11.5% in the navigated and of 8.9% in the fluoroscopy group. Verbeek et al. [17] reported similar data in 2016 (17% misplaced screws in the navigated technique vs 16% misplaced screws in the fluoroscopy technique). Some authors even state that navigation comes along with a significant higher risk for misplacement of the screws and subsequent neurological complications [18]. All these investigations give no information why screw misplacement occurred when navigation was applied. In our investigation we found a rate of misplaced screws in the fluoroscopy group of 10.9% and of 0% in the navigation group. These results are comparable with the data presented by Peng et al. in 2013 [9] (0% of misplaced screws) or by Boudissa et al. in 2022 [19] (2.2% of misplaced screws). Thus, in our point of view navigation leads to a clear benefit in positioning of the screws when it is applied. It has to be mentioned, that we did not create subgroups according to different sacral anatomies in both study groups. This could have caused a bias in our results with regard to accuracy of screw placement. However, for every included patient the preoperative CT scan was analyzed to plan surgery, different sacral anatomy was taking into account in this planning. Thus, sacral anatomy was precisely known during surgery and might not have influenced the rate of misplaced screws. Independent of this topic differences in sacral anatomy did not affect time and dose of radiation.

The effect of applying navigation in ISS placement with regard to duration of surgery is not clear in the current literature. Some authors state that there is no difference in duration of surgery [20], while other authors describe an even longer duration of surgery when navigation is applied [16, 19]. In our investigation we found a significant shorter duration of surgery when navigation is used. This is comparable with the results presented by Zhao et al. in 2019 [20] and Madeja et al. in 2022 [22]. It has to be mentioned that in all investigations dealing with this topic there is a wide range of the reported durations of surgery weather it is performed by navigation or by fluoroscopy. Implementation of new techniques (a.e. use of navigation instead of fluoroscopy) always comes along with a learning curve [23]. This learning curve may influence the results of the duration of surgery. However, according to our results, after standardization and completing the learning curve for use of navigation in ISS placement it leads to a reduced duration of surgery.

It has to be discussed, that patients from the fluoroscopic group were included during a longer period than patients from the navigated group; this might have let to more inhomogeneous results in the fluoroscopic group and maybe influenced the results of our investigation. But, due to the fact, that all surgeons who performed surgery had a lot of experience in placement of ISS even before data collection was started in the fluoroscopic group, the technique of ISS placement did not change during the time and in every patient the same C-arm was used, we think the longer period of including patients in the fluoroscopic group did not let to an bias in our results.

Dose of radiation and duration of radiation use is discussed controversy in the current literature. Most authors report a reduced dose and duration of radiation when navigation is applied in ISS placement [14, 15, 20–22]. These results are comparable with the results reported an exposure to radiation in the navigated placement of pedicle screws [10, 11]. The results of our investigation (significant reduced duration of radiation use and emitted dose of radiation if ISS placement was performed with navigation) confirm these results. However, there are some authors who state, that navigation in ISS placement leads to the same [16] or an even higher [9, 20] exposure to radiation. A possible explanation for these differing results may be, that there are many different intraoperative imaging modalities (a.e C-Arm, O-Arm, intraoperative CT) each with different rates of exposure to radiation. Furthermore, there are many different navigation systems and techniques available which also influence the exposure to radiation. Thus, according to our results, we can only state that ISS placement with navigation leads to a reduced exposure to radiation when a C-Arm is applied for imaging and navigation is applied in the way we explained it in the methods section.

The influence of use of navigation with regard to exposure to radiation for the surgical team is not discussed in the current literature. In our investigation we were not able to measure the exposure to radiation for the surgical team directly. We only were able to estimate the reduced time in the operation room during application of radiation for the surgical team when navigation is applied. However, it seems clear, that due to the fact that navigation comes along with a reduced need of conventional imaging, radiation exposure for the surgical team is reduced, because they leave the operation theater during the scan.

A limitation of our investigation might be that the group of patients treated by ISS placement by fluoroscopy was included retrospectively. Thus, exposure to radiation could not be measured directly at the patient and had to be extrapolated by the dose area product emitted by the C-Arm. Furthermore, there was no official study protocol for the treatment of the retrospectively included patients. However, due to a standard operating procedure in our department for the treatment of sacral fractures the treatment of the patients was comparable without an official study protocol. The relatively low number of included patients’ needs to be mentioned as a further limitation of our investigation. But, due to the fact, that our presented results all showed statistically high significant differences we do not think our investigation is underpowered due to the relatively low number of included patients’.

## Conclusion

Based on these results MIS ISS placement guided by navigation seems to be a safe procedure. It leads to a reduced exposure to radiation for the patient and the surgical team. Furthermore, MIS ISS placement leads to a higher accuracy in placement of the screws and to a reduction of duration of the surgery.

## Data Availability

The datasets generated during and/or analyzed during the current study are not publicly available due to data protection but are available from the corresponding author on reasonable request.

## Abbreviations

ISS: Iliosacral screw
MIS: Minimal invasive procedure
DAP: Dose area product
SEM: Standard error of mean
r: Correlation
p: Level of significance
